# Current War in Ukraine: Lessons from the Impact of War on Combatants’ Mental Health during the Last Decade

**DOI:** 10.3390/ijerph191710536

**Published:** 2022-08-24

**Authors:** Andriy Haydabrus, Mikel Santana-Santana, Yuriy Lazarenko, Lydia Giménez-Llort

**Affiliations:** 1 Department of Neurology, Psychiatry, Narcology and Medical Psychology, School of Medicine, V.N. Karazin Kharkiv National University, 61022 Kharkiv, Ukraine; 2 Department of Psychiatry and Forensic Medicine, School of Medicine, Universitat Autònoma de Barcelona, 08193 Barcelona, Spain; 3 Institut de Neurociències, Universitat Autònoma de Barcelona, 08193 Barcelona, Spain; 4 Military Medical Clinical Center of the Northern Region, 61000 Kharkiv, Ukraine

**Keywords:** mental health, war, stress, Ukraine, soldiers, ICD-10, anxiety, psychoactive drug use, military rank

## Abstract

Ukrainian Military Hospital retrospective analysis during a decade of conflicts (3995 records) unveils specific mental health ICD-10-CM distribution per rank and the long-lasting impact of active conflict or trench warfare. Most hospitalizations in all years of observation were among soldiers. Anxiety-related disorders have been present since ‘peacetime’, mainly among professional soldiers and high ranks, pointing to the need for rank-tailored psychological training in skills to reduce the anxiety burden. High frequency of psychoactive substance use emerged with acute conflicts and in nonprofessional soldiers during wartime. This dictates the need to strengthen the selection of military personnel, considering the tendency to addiction. Military operations multiply the hospitalizations in psychiatric hospitals. The data warn about a ‘need for free beds effect’, which is worse for soldiers. This is relevant to estimating and planning the need for hospital resources for the current situation where the general population has been recruited for defense. In the current war, tightening the rules of sobriety in units and up to a ban on the sale of alcoholic beverages in areas where hostilities are taking place is recommended. The specific impact on nonprofessional soldiers is relevant to the current war, with the general population of Ukraine recruited for defense and combat.

## 1. Introduction

Since 24 February 2022, the Russian war in Ukraine has become the most critical international geopolitical crisis, with high human costs, including civilian casualties, mass migration, and ripple effects having a global impact [1,2,3,4,5,6]. However, the threat and preservation of the territorial integrity of Ukraine are not new issues. During the last decade, peacetime (Peace, until 2013) was disrupted by active hostility (AH, 2014–2015) and trench warfare (TW, 2016–2021) [1].

War and/or severe armed conflicts exert acute and chronic impacts on mental health, but they are challenging to measure during battle [1,7,8]. In the current scenario, the war may be a substrate for mental health disorders, especially worrisome since, today, the large-scale conflict has demanded the recruitment of adult civilians to defend and fight alongside armed forces troops. One of the first studies just reported the impact of the war on the mental and emotional well-being of Ukrainian civilians—university students and personnel of four heavily bombed universities [9]. Active hostility exerts a severe impact not just on the population of sides of the conflict. A wave of mental health consequences of the current Russian war in Ukraine in non-directly exposed populations has also been reported in other countries [10,11].

This communication presents a retrospective cross-sectional analysis of 3,995 admissions and mental health reports ([variables: military rank, days of hospitalization, and mental health disorders according to ICD-10-CM [12]) in a Ukrainian Military Hospital Mental Health inpatient setting during the last decade of conflicts. The analysis aimed to (1) unveil the impact of those conflicts on the mental health of the army and (2) help us to anticipate risk factors (ranks, time period) and need for resources (admissions and days of hospitalization per time period, rank and disease) as well as estimate the current impact on mental health, particularly on civilians mobilized in combat, to hamper the effects and tailor interventions.

## 2. Materials and Methods

### 2.1. Database and Study Design

A retrospective cross-sectional analysis of an anonymized part of the internal database of the Military Medical Center of the Northern Region, Kharkiv, Ukraine, that can be used for scientific purposes was performed. The database set of the last decade (2012–2021) included 3995 anonymized records, year of admission, military rank, days of hospitalization recorded on a regular basis, as well as the mental health disorders as assessed by first and third authors after admissions and according to ICD-10-CM (F01–F99, Mental, Behavioral, and Neurodevelopmental disorders) [12].

According to the chronology of the events in the eastern part of Ukraine during the last decade [1], the timeline was divided into three time periods: Peacetime (2012–2013), Active Hostility (AH, Donbas conflict: April to September 2014; January–February 2015; since February 2015, positional battles went on) and Trench Warfare (TW; TW1, 2016–2017; TW2, 2018–2019; TW3, 2019–2021).

### 2.2. Statistical Analysis

Data are expressed as the frequency (%), fold-increase, or mean ± SEM. Chi-square analysis and ANOVA with Bonferroni post hoc correction were performed with Jamovi (Sidney, Australia) [13]. In a secondary analysis per rank and war period, TW3 was excluded since, during the COVID-19 outbreak, the data on ranks were incomplete, and the results showed convergence on TW2. For particular Chi-square comparisons, GraphPad QuickCalcs Web site: https://www.graphpad.com/quickcalcs/contingency1/ San Diego, CA, USA (accessed on 25 June 2022) by Dotmatics was used. Figures were generated with GraphPad and edited in PowerPoint. Chi-square, in all cases, *p* < 0.05 was considered statistically significant.

## 3. Results

### 3.1. Anxiety Disorders Were Already Present in Peacetime, While a High Frequency of Psychoactive Substance Use Emerged with Acute Conflicts

**Figure 1:**
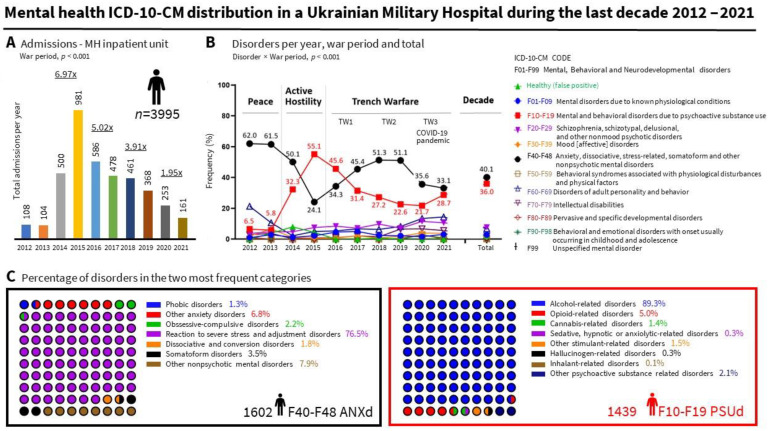
Mental health distribution in a Ukrainian Military Hospital during the last decade. (**A**) Temporal distribution of admissions in a mental health (MH) inpatient unit. (**B**) Frequency (%) of disorders per year, war period, and decade. (**C**) Frequencies of disorders in the two most frequent categories. ANXd: Anxiety, dissociative, stress-related, somatoform and other nonpsychotic mental disorders; PSUd; Mental and behavioral disorders due to psychoactive substance use. Statistics (see depicted in the insets): Frequencies (%), Chi-square.

During the last decade, the temporal distribution of admissions (Figure 1A) showed a 6.97 (AH) and 3.62 (TW) [5.02 (TW1, 2016–2017), 3.91 (TW2, 2018–2019), and 1.95 (TW3, 2020–2021)] fold increase per year compared to peacetime. The most frequent mental health problems (Figure 1B), accounting for 76.1% of cases, were ‘anxiety, dissociative, stress-related, somatoform and other nonpsychotic disorders’ (F40-F48, ANXd, 40.1%, 1602/3995) and ‘mental and behavioral disorders due to psychoactive substance use’ (F10–F19, PSUd, 36.0%, 1439/3995). ‘Reaction to severe stress and adjustment disorders (F43, 76.5%, 1225/1602) and ‘Alcohol-related disorders (F10, 89.3%, 1285/1439) were the predominant mental health disorders, respectively (Figure 1C). Less than 10% of cases were diagnosed with ‘schizophrenia, schizotypal, delusional, and other nonmood psychotic disorders’ (F20–F29, 7.7%, 306/3995) or ‘disorders of adult personality and behavior’ (F60–F69, 6.8%, 273/3995).

### 3.2. An Increase in Psychological Stress Symptoms Could Be a Confounding Factor in Admissions Diagnosed as ‘Healthy’

Interestingly, 2.3% (90/3995) of cases admitted to the psychiatric inpatient unit were diagnosed as ‘mentally healthy’. When analyzed per period [peace, 3.8% (4/212); active hostility, 12.4% (83/1478); trench warfare, 0.001% (3/2305)], the results suggested that an increase in psychological stress symptoms could work as a confounding factor. This false-positive effect could also explain the higher increase in active hostility. Military personnel suffers from a set of disorders and psychological consequences caused by extreme trauma, which impacts the individuals leading to a general feeling of instability and promoting more anxiety [14]. These stress response symptoms could be misdiagnosed as symptoms of a disease.

### 3.3. The ICD-10 Category Depended on the War Period 

A secondary analysis indicated that the ICD-10 category depended on the war period (Figure 1B), with peacetime to TW2 accounting for 90% (3582/3995) of cases. ‘ANXd’ were the main mental health problem in any period, with 61.8% (131/212) of cases occurring in peacetime. Their frequency decreased in active hostility (32.9%, 486/1478) and TW1 (39.3%, 417/1064) and increased in TW2 (51.2%, 424/828). This was not due to fewer admissions with ANXd but an increased number with other pathologies (mainly PSUd). PSUd, residual in peacetime [6.1%, (13/212)], reached their peak in active hostility (47.4%, 700/1478), with 97.9% (685/700) of ‘Alcohol-related disorders’ as the cause of these admissions (Figure 1C), which could agree with the use of alcohol serving as a coping mechanism in front traumatic events [15]. We can also speculate that people that use alcohol might be those with an addiction. Thus, alcohol consumption may not only be related to dealing with stress and symptoms of psychological trauma but also to addiction. Military service in a war zone has a relatively modest long-term effect on the alcohol drinking patterns of male veterans [16]. In trench warfare, PSUd decreased (TW1, 39.2%, 417/1064; TW2, 25.1%, 208/828) but still occupied a large proportion, with a decrease more remarkable than that caused by the decrease in the sample.

‘Schizotypical disorders’ experienced a gradual increase to their peak at TW2 (8.6%, 71/828), being twice as frequent as in peacetime (4.2%, 9/212). ‘Mood (affective) disorders’ (Peace, 4.2%, 9/212) reduced their frequency during conflicts (range: 0.8–1.3%). Despite the other categories being very residual, some showed conspicuous changes. ‘Personality disorders’ peaked in peacetime (16.3%, 34/212) and decreased afterward. ‘Mental health disorders due to known physiological conditions’ (F01-F09, 2.8%, 111/3995), relatively stable and with a peak in TW1 (4.8%, 51/1064), showed a 7- (AH), 12.75- (TW1), and 4.75- (TW2) fold increase compared to peacetime (4/212). ‘Behavioral syndromes associated with physiological disturbances and physical factors’, ‘Pervasive’, and ‘Childhood disorders’ were stable and residual. ‘Intellectual disabilities’ were relatively stable (range: 0.7–3.7%).

### 3.4. The Dramatic Increase in Admissions and the Specific Mental Health Profiles per Rank Put the Hospital’s Efficiency at Risk

If we could protect hospitals from the waging of war [17], their efficiency would still be compromised by the dramatic increase in admissions and restrictions on human and medical resources.

**Figure 2:**
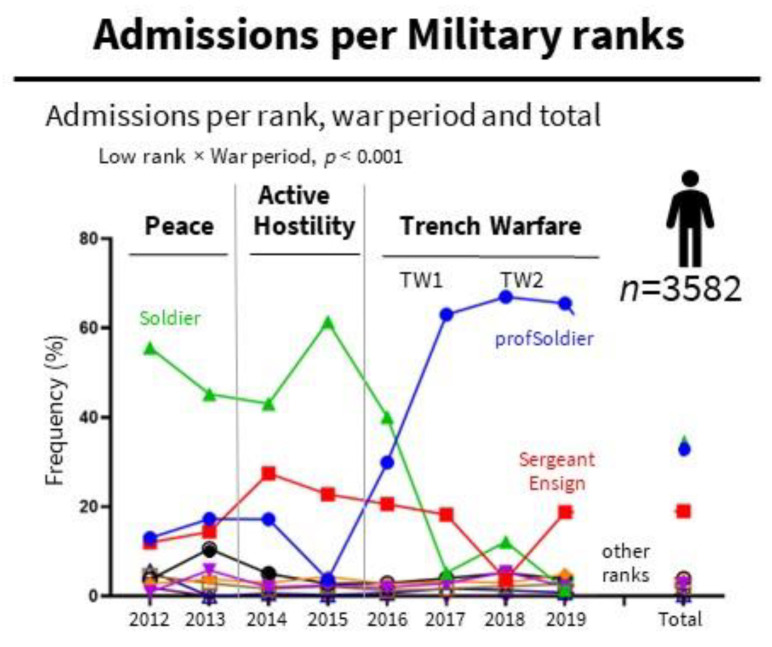
Admissions per Military ranks. Admissions per rank, war period and total ANXd: Anxiety, dissociative, stress-related, somatoform and other nonpsychotic mental disorders; PSUd; Mental and behavioral disorders due to psychoactive substance use. Statistics (see depicted in the inset): Frequencies (%), Chi-square.

The admissions (Figure 2) of high ranks (Major, Lieutenant Colonel, and Colonel) were relatively stable over time. ANXd were the most frequent mental health disorders and increased with rank [Major (63%, 46/73), Lieutenant Colonel (72.7%, 24/33), and Colonel (100%, 2/2)]. Despite their small sample size, the analysis of high ranks is of interest due to their military roles. 

In contrast, admissions of low ranks (Soldiers; professional Soldiers: Contract Service Soldiers; and Sergeant-Ensign) depended on the war period. Soldiers showed a peak in peacetime (50.5%, 107/212) and active hostility (55.2%, 816/1478) and decreased in trench warfare (TW1 24.4%, 260/1064; TW2, 7.4%, 61/828). In contrast, professional soldiers (Peace, 15.1%, 32/212; AH, 8.3%, 122/1478) peaked at trench warfare (TW1, 44.7%, 476/1064; TW2, 66.3%, 549/828). With lower frequency, Sergeant-Ensign (Peace, 13.2%, 28/212) peaked in active hostility (24.4%, 360/1478) and trench warfare (TW1, 19.5%, 208/1064; TW2, 10.4%, 86/828).

### 3.5. The Specific Impact on PSUd in Nonprofessional Soldiers Is Relevant to the Current War, with the General Population of Ukraine Recruited for Defense and Combat

**Figure 3:**
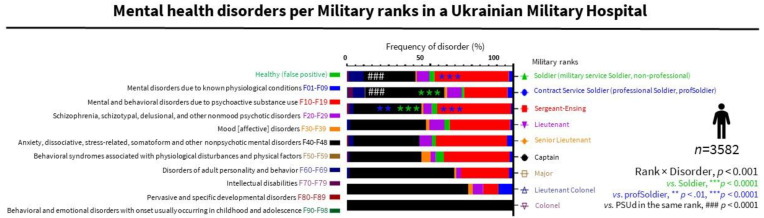
Mental health disorders per military ranks. Left axis: ICD-10-CM categories. Right axis: Military ranks. Statistics: Frequencies (%), Chi-square (see depicted using colors, in the inset).

In addition, a rank × disorder effect (Figure 3) indicated that soldiers showed more PSUd (44.7%, 556/1244) than ANXd (31.5%, 392/1244) and professional soldiers (PSUd, 26.1%, 308/1179). In contrast, professional soldiers showed more ANXd (47.5%, 560/1179) than PSUd and compared to soldiers. These results suggest different vulnerability and coping strategies among ranks with implications for designing tailored interventions and managing hospital resources. Additionally, they warn of the impact of the current war on civilians recruited or volunteering as soldiers.

### 3.6. A ‘Need for Free Beds Effect’ Could Explain Shorter Hospital Stays during Active Conflict and Anticipate Similar Phenomena in the Current War 

**Figure 4:**
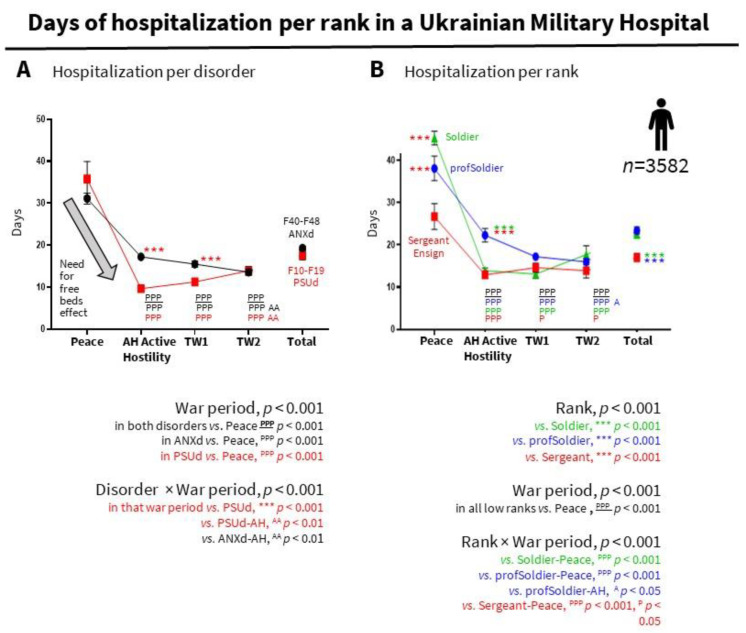
Days of hospitalization. (**A**) Days of hospitalization per disorder, and (**B**) per military rank. ANXd: Anxiety, dissociative, stress-related, somatoform and other nonpsychotic mental disorders; PSUd; Mental and behavioral disorders due to psychoactive substance use. Statistics (see depicted in the insets): Frequencies (%), Chi-square; and Mean ± SEM, ANOVA with post hoc Bonferroni.

Hospital stays for people with ANXd or PSUd lasted at least one month in peacetime but significantly decreased in war periods (Figure 4). This could be explained by a ‘need for free beds effect’ (Figure 4A) and the distribution of admissions by ranks (Figure 2 and Figure 4B). In addition, in active hostility and trench warfare TW1, people with PSUd spent fewer days than those with ANXd, probably due to a high frequency of ‘acute alcohol-related disorders’ among PSUd in these periods (97.9% and 84.5%, respectively). After active hostility, PSUd increased while ANXd decreased. 

Sergeant-Ensigns had shorter hospitalization than nonprofessional and professional soldiers. Hospitalization was also analyzed in the three lowest ranks since they were the most significant part of the sample size (Figure 4B). The mean showed no differences between the two soldier categories, but both had longer hospitalization times than Sergeants-Ensigns. Significant effects of the war period, rank, and interaction were also found, with a drastic reduction after peacetime that was more substantial in soldiers. The temporal curves mimicked those per disorder (Figure 4A) and would agree with a higher frequency of PSUd in soldiers and ANXd in the other two professionalized ranks. This may indicate that the need for empty beds has contributed to a reduction in the length of treatment, as well as an increase in the number of military personnel with anxiety and intoxications who did not require long-term treatment.

## 4. Discussion

In the previous section, subheadings have highlighted the main findings, and specific discussion has been added to each of the results. Further aspects to discuss are presented here:

War to defend the state has its own characteristics. In the literature, we have only seen studies in the professional military. The most common disorders among active military personnel are depression and PTSD [18]. In contrast, in our work, we have also observed mental health disorders among professional military data. Two categories of mental disorders, namely the PSUd and ANXd, were the most prevalent and were mainly found among soldiers.

It is important to note that, similarly to the current situation, during the last decade in Ukraine, many civilian populations were recruited for defense and combat. Therefore, the hospital units received two distinct categories of soldiers, nonprofessional soldiers, and contract service (professional) soldiers. The retrospective analysis unveiled that in each group of soldiers (nonprofessional and professional), the proportions of the most prevalent disorders, PSUd and ANXd differed. Our study is supported by current data from survey results; the study in the four Ukrainian universities—with a 1:6 male:female ratio—reports important psycho-emotional alterations in most of the participants (97.85), shown as exhaustion (86.7%), depression (84.3%), nervousness (84.4%), anger (76.9%) and loneliness (51.8%), with age/status (students more than personnel) and gender (females more than males) effects. The use of substances (males more than females) (i.e., tobacco, alcohol, pain relievers, and sedatives) and unhealthy food has increased as well as loneliness associated with fear, burnout and lower resilience. Furthermore, civil status influenced loneliness, with unmarried participants feeling more lonely than unmarried. As we can see, the symptoms of mental disorders were associated with the use of drugs among civilians.

Alcohol addiction is common among military personnel, but this is due to the presence of anxiety disorders, symptoms of stress, and the presence of symptoms of PTSD. In the category of nonprofessional soldiers, PSUd and, in particular, alcohol dependence occurred as an independent disorder. Thus, the retrospective analysis indicated the predisposition of the nonprofessional soldier to alcohol dependence. This may be a consequence of insufficient military service screening during hostilities outbreaks [19].

Given the substantial cost of alcohol misuse, it is imperative to examine factors that may contribute to problematic drinking so that interventions can be employed to address this issue within the military. Military-related traumatic stress seems to elevate the risk of individuals misusing alcohol. The co-occurrence of posttraumatic psychiatric disorders seems to play a major explanatory role in the association between military stress and alcohol misuse. Screening and intervention for alcohol misuse, particularly following exposure to military-related trauma, are clearly needed, as are integrated treatments that address conjoined alcohol and PTSD problems [20].

Because the available data only detect the primary underlying disease, we cannot determine the level of relationship between PSUd (alcohol abuse) and ANXd (anxiety). However, in this respect, the recent study in four Ukrainian universities in the eye of the storm [9] has reported two significative data in this respect: the level of substance use by their students/personnel before the war (69.9%, alcohol > tobacco > pain killers > hypnosedatives)—higher in males than females—has increased during the war (10 to 19%, alcohol > hynosedatives > tobacco > pain killers) and is associated with lower resilience; higher fear, burnout, and loneliness. Therefore, given that in our retrospective study the group of nonprofessional soldiers recorded more alcohol abuse, we can offer less discipline among this contingent and a greater propensity for dependence. 

Therefore, the relationship between addictions and the regulation/dysregulation of emotions shown to play an important role both in war and peace times in other conflicts [21] should be considered for tailored interventions since adaptive emotional regulation strategies decrease the experience of negative emotions [11]. In particular, the Italian study on the role of emotion regulation on aggressive responses related to the current Russian–Ukrainian conflict has shown the negative effect of cognitive reappraisal of emotions [11]. 

Professional military officers suffered mainly from neurotic disorders and anxiety during their current service. If these disorders are not treated, they will be the basis for the future development of PTSD, adjustment disorders, anxiety disorders, and depressive disorders [22]. Not only treatment but also prevention of anxiety disorders is important to reduce the burden on hospital staff.

Some of the limitations of this retrospective study are: The data on a masculinized military structure limit the analysis under the gender perspective needed in the current war, where female civilians have also joined the combat. The university study [9] already indicates the relevance of gender effects on mental health. The transversal design cannot provide clues on the long-term effects on an individual base, i.e., the subsequent increase in alcohol dependence and vulnerability to mental health disorders described in veterans [8,14,23].

## 5. Conclusions

The three main conclusions and implications for the current war can be summarized as:

1. The dominance of ANXd, mainly among professional soldiers and high ranks, points to the need for rank-tailored psychological training in skills to reduce the ANXd burden.
2. The large number of PSUd in nonprofessional soldiers during wartime dictates the need to strengthen the selection of military personnel, taking into account the tendency to addiction. In the current war, tightening the rules of sobriety in units and up to a ban on the sale of alcoholic beverages in areas where hostilities are taking place is recommended.
3. Military operations multiply the hospitalizations in psychiatric hospitals. Admissions are heterogeneous and depend on the military rank (soldiers and professional soldiers spend more time than other low ranks). This is relevant to estimating and planning the need for hospital resources for the current situation where the general population has been recruited for defence and combat.

## Data Availability

The dataset is available upon request.
